# SURGICAL TREATMENT OF CLAVICLE FRACTURES: A DESCRIPTIVE ANALYSIS OF 88 CASES

**DOI:** 10.1590/1413-785220233103e264456

**Published:** 2023-07-17

**Authors:** LEANDRO MARANO RODRIGUES, RAFAEL SAMPAIO OLIVEIRA, LERUD FROSI NUNES, FERNANDO FURTADO LÁZARO, ANGELO TON, ANDERSON DENADAI

**Affiliations:** 1 Santa Casa de Misericordia de Vitoria, Departamento de Cirurgia de Ombro e Cotovelo, Vitoria, ES, Brazil.; 2 Vitoria Apart Hospital, Departamento de Cirurgia de Ombro e Cotovelo, Serra, ES, Brazil.; 3 Vitoria Apart Hospital, Instituto de Ortopedia e Traumatologia, Serra, ES, Brazil.

**Keywords:** Fracture Fixation, Clavicle, Epidemiology, Fixação de Fratura, Clavícula, Epidemiologia

## Abstract

Clavicle fractures are one of the most common types of bone injuries in adults. Recently, the treatment protocol for these fractures has undergone changes.

**Objective:**

To better understand the epidemiological and behavioral profile of these lesions when they require surgical treatment.

**Methods:**

This is an analysis of a series of cases. Our sample included individuals undergoing surgical treatment for clavicle fractures.

**Results:**

In total, 88 subjects fulfilled the predetermined criteria. Among these, 75 (85.22%) were male; automobile accidents corresponded to the largest etiological group, reaching 48% of prevalence; there was a slight predominance of the right side, totaling 45 cases (51%); most fractures were classified as Allman type I; an infection rate was observed in 1.13% of the cases; and the development of pseudarthrosis was identified in 2.27% of the patients.

**Conclusion:**

The incidence of clavicle fracture is higher in young men, mainly caused by car accidents, being mostly located in the middle third. No statistical significance was found between the synthesis material data and the postoperative complication rate, revealing the absence of risk superiority between the different types of surgical approaches used. ***Level of Evidence IV, Case Series.***

## RESUMO

As fraturas de clavícula consistem em um dos tipos mais comuns de lesões ósseas na população adulta. O protocolo de tratamento dessas fraturas tem sofrido modificações.

**Objetivo:**

Compreender melhor o perfil epidemiológico e comportamental das lesões de clavícula submetidas a tratamento cirúrgico.

**Métodos:**

Análise de uma série de casos, cuja amostragem incluiu indivíduos submetidos à abordagem cirúrgica de fratura de clavícula.

**Resultados:**

No total, 88 indivíduos preencheram os critérios pré-determinados. Dentre estes, 75 (85,22%) eram do sexo masculino; o acidente automobilístico correspondeu ao maior grupo etiológico (48%); houve ligeira predominância do lado direito, totalizando 45 casos (51%); a maior parte das fraturas foi classificada como Allman tipo I; observou-se uma taxa de infecção em 1,13% dos casos; e o desenvolvimento de pseudoartrose foi identificado em 2,27% dos pacientes.

**Conclusão:**

A incidência de fratura de clavícula é maior em homens jovens, ocasionada principalmente por acidentes automobilísticos, sendo, na maioria dos casos, localizada no terço médio. Não foi encontrada significância estatística entre os dados do material de síntese e o índice de complicação pós-operatória, revelando a ausência de superioridade de risco entre os diferentes tipos de abordagem cirúrgica. **Nível de Evidência IV, Série de Casos.**

## INTRODUCTION

Clavicle fractures are one of the most common types of bone injuries in adults, accounting for about 2.6% of all fractures and 44.1% of fractures among the shoulder bones.^1^ ^)-(^ ^3^ Recently, the treatment protocol for clavicle fractures has undergone changes due to the evident superiority of surgeries regarding the development of functional sequelae. ^(^ ^2^ Moreover, several studies have reported an increase in the prevalence of clavicle fractures in recent decades and it is believed that this raise is mainly due to the increase in the practice of sports and in the use of motorcycle vehicles, ^(^ ^3^ valuing the focus on the study of the treatment of these injuries.

The objective of this retrospective study is to better understand the epidemiological and behavioral profile of postoperative clavicle fractures subjected to surgery for a period of six years in a tertiary hospital.

## METHODS

This is an observational and cross-sectional study, with a sample including both males and females, without age restriction, who have been subjected to the surgical approach of clavicle fracture from February 2014 to May 2020 in a specialized orthopedic center.

The research was approved by the Research Ethics Committee registered in the Brazil Platform under the CAAE no. 51097521.4.0000.5073, following the guidelines of resolution 446/12 of the National Health Council, Brazilian Ministry of Health.

The inclusion criteria attributed to the research were: (1) use of surgical coding 3.07.17.10-8 (Brazilian Hierarchical Classification of Medical Procedures - CBHPM) with the term “fracture and/or dislocations and/or avulsions - surgical treatment” in medical records and (2) date of care from February 2014 to May 2020. The exclusion criteria were: (1) association and/or reference to surgical approaches involving distinct structures and (2) absence of complete data in medical records.

Regarding the surgical technique used, the patients were stationed in a beach chair position, as Hoppenfeld^4^ indicates, and the palpation of the clavicle was performed to locate the focus of the fracture and determine the extent of the incision. Immediately after, an S-shaped anterosuperior incision was made, starting at the medial extremity, followed by a dissection by planes of the soft parts of the subcutaneous face of the clavicle in the subperiosteal plane to expose the lesion. After this step, reduction and internal fixation (absolute or relative, depending on the fracture profile) were performed with the use of plates and screws. In cases of Allman II fracture, fixation was performed with Kirschner wires in association with anchor fixation in the coracoid or subcoracoid ligature.

In total, 88 individuals met the predetermined criteria and were included in the research. The clinical variables analyzed were: (1) age; (2) sex; (3) affected side; (4) mechanism of trauma; (5) fracture classification according to Allman’s description; (6) synthesis material used in surgical treatment; (7) need for reoperation; (8) development of operative complications; and (9) associated lesions.

Demographic data were collected, organized, and tabulated using the Excel software program. All indices were expressed as mean ± standard deviation. P-values < 0.05 were considered statistically significant. Statistical analysis was performed using the GraphPadPrism program, version 7.0.

All participants signed an informed consent form. The research was approved by the Research Ethics Committee on September 10, 2021, according to substantiated opinion number 4,963,234.

## RESULTS

Among the individuals included in the study, 13 (14.77%) were female and 75 (85.22%) were male, with ages ranging from 11 to 64 years (mean of 34.94 years) at the time of surgery.

Among the types of trauma mechanism identified in the study, automobile accidents corresponded to the largest etiological group, reaching 48% prevalence, while 26% were attributed to sports-related trauma, 10% to falls from one’s own height, and 8% to falls from a bicycle. As for the affected side, there was a slight predominance of the right side, totaling 45 cases (51%). Most fractures were classified as Allman type I, corresponding to 72 fractures (81.81%), while 16 fractures (18.18%) were classified as Allman type II. There were no fractures classified as Allman type III.

Table 1 shows the demographic clinical characteristics of the analyzed participants segregated by sex. No significant differences were observed between the groups regarding the frequency of the etiologies ‘automobile accident’ and ‘fall from one’s own height’ as trauma mechanisms, maintaining the etiologies ‘sports-related traumas’ and ‘bicycle falls’ exclusively in males. There were also no differences between the groups regarding the affected side, with a predominance, in both, of traumas involving the right shoulder and the classification of the injuries according to the Allman model.

**Table 1:** Main clinical and demographic characteristics of patients.

Characteristic	Female (n = 13)	Male (n = 75)	***p***
**Age (years)**	34.5 ± 2.0	31.3 ± 4	*0.93*
**Trauma mechanism (%)**			
Car accident	48.0 ± 0.5	44.1 ± 0.8	*0.49*
Sports-related traumas	-	9.3 ± 1.0	
Fall from one's own height	49.3 ± 0.7	46.8 ± 1.0	*0.85*
Bicycle fall	-	3.3 ± 0.5	
**Affected side**			
Right	57.3 ± 0.6	53.8 ± 0.82	*0.51*
Left	42.6 ± 0.7	46.15 ± 0.7	*0.69*
**Lesion classification**			
Allman Type I	15.3 ± 0.6	17.3 ± 0.5	*0.68*
Allman Type II	84.7 ± 0.8	82.6 ± 0.9	*0.54*

*Values were expressed as mean ± standard deviation.

The synthesis materials used in the osteosynthesis of fractures were 15 reconstruction plates, 46 locking clavicle plates, 11 locking reconstruction plates, 1 dynamic compression plate (DCP), 1 locking T-plate, 1 distal clavicle plate, 4 Kirschner wires, 6 Kirschner wires with anchor, and 3 Kirschner wires with ligature.

During clinical follow-up of patients undergoing surgical treatment, it was necessary to remove the synthesis material from 7 patients (7.95%). One patient (1.13%) presented evidence of postoperative infection and was subjected to the removal of the Kirschner wire with anchor; 2 individuals with a history of osteosynthesis using a locking clavicle plate evolved to pseudarthrosis (2.27%); and 4 patients opted for the removal of the material (2 locking clavicle plates, 1 DCP plate, and 1 reconstruction plate) due to local discomfort. None of the patients presented loosening of material or postoperative brachial plexus injury.

Finally, regarding associated injuries, 1 (1.13%) patient had open clavicle fracture and 2 (2.7%) presented cases with brachial plexus injury in the preoperative period.

## DISCUSSION

The results obtained in our analysis are corroborated by Herteleer et al., ^(^ ^5^ which reported the predominance of males as the most affected by this type of injury in 2017. Regarding average age, several studies have identified a bimodal distribution, ^(^ ^1^ ^),(^ ^2^ ^),(^ ^5^ while Kihlström et al. ^(^ ^1^ observed an overall average of 48 years, affecting males at a younger mean age (43 years) and females at an older age (59 years). Conversely, in our study, we observed a general average of 34.94 years, with a average age fractionated by sex of 35.6 years in females and 58.04 years in males, which can be justified by the cultural differences in our population and the high rates of automobile accidents in younger patients.

Regarding affected side, our findings agree with the literature. Souza et al. ^(^ ^6^ analyzed 26 osteosyntheses in 25 patients with midshaft clavicle fracture, occurring in 50% of the cases on the right, with a mean similar to that defined in our study. 

Regarding fracture classification, the Allman classification was used as a descriptive criterion. Typically, midshaft clavicle fractures (Allman type I) are the most frequent, accounting for about 80% of cases, occurring mostly in young patients. Fractures located at the lateral end of the clavicle (type II) correspond to approximately 15%-25%, and, to a lesser extent, about 5% occur in the proximal third (type III). ^(^ ^7^ Regarding the data of this study, a higher incidence of Allman type I fractures was observed (81.81%), corroborating the literature.

Regarding mechanism of the trauma that caused the fracture, a discrepancy of probable cultural origin was observed. Our study showed a higher occurrence of clavicle fracture after motorcycle accidents (48%) and sports-related traumas (26%), while the European population-based articles, such as the one by Herteller et al., ^(^ ^5^ reported that the most common trauma mechanism was bicycle accidents (35.3%-20.5%) and low-energy falls (34.1%-14.5 %).^1^ ^),(^ ^5^

Regarding postoperative complications, in our follow-up, a rate of infection related to the surgical site was observed in 1.13% of the cases, corresponding to an index lower than that described by Leroux et al., ^(^ ^8^ who found a percentage of 2.6%. The development of pseudarthrosis was identified in 2.27% of the cases studied in our database, in a proportion similar to that defined by Altamimi et al. ^(^ ^9^ and Zlowodski et al. ^(^ ^10^ Finally, no literature data were found that corroborate or contradict our statistical indices regarding surgical reoperation to remove synthesis material due to complaints of discomfort. 

After statistical analysis of correlation, no statistical significance was found between the data of synthesis material and postoperative complication index, revealing the absence of superiority of risk between the different types of surgical approach used in our research. Similarly, Ashman et al. ^(^ ^11^ compared the postoperative results between fixation with reconstruction plate and compression and found no difference in the clinical outcome. Despite the neutrality of the negative results, after extensive analysis in the follow-up period, we believe that the best synthesis materials for the surgical treatment of clavicle fractures are the reconstruction plate and the locking clavicle plate, considering the bone anatomy and the possibility of molding the reconstruction plates according to the individual particularity and the pre-molding of the locking clavicle plate, which provides an anatomy similar in both appearance and physiological aspect.

## CONCLUSION

This study showed that the incidence of clavicle fracture is higher in young men, is mostly caused mostly by automobile accidents, and is most often located in the middle third of the clavicle.

We also observed a low rate of postoperative complications and satisfactory functional results considering the various surgical fixation materials used, with negative correlative analysis when comparing the type of approach and clinical outcomes. Thus, we recommend for detailed analyses to be conducted on the synthesis material used, considering the importance of using a low profile and molded plate, reducing the need to remove the material due to discomfort.

The data of this analysis present sampling bias since it was limited to a private tertiary hospital. Thus, we conclude that efforts should be directed in the design of large, controlled, and randomized population-based studies, to further extrapolate the results found.
